# Laparoscopic resection for retroperitoneum ganglioneuroma with Supine hypotension syndrome

**DOI:** 10.1186/s40792-024-01992-w

**Published:** 2024-08-20

**Authors:** Yu Sugai, Masaya Yamoto, Juma Obayashi, Takafumi Tsukui, Akiyoshi Nomura, Hiromu Miyake, Koji Fukumoto, Sung-Hae Kim, Daijiro Sato, Hideto Iwafuchi

**Affiliations:** 1 Department of Pediatric Surgery, Shizuoka Children’s Hospital, 860 Urushiyama, Aoi-Ku, Shizuoka, 420-8660 Japan; 2 Department of Cardiology, Shizuoka Children’s Hospital, 860 Urushiyama, Aoi-Ku, Shizuoka, 420-8660 Japan; 3 Department of Pathology, Shizuoka Children’s Hospital, 860 Urushiyama, Aoi-Ku, Shizuoka, 420-8660 Japan

**Keywords:** Retroperitoneum, Retroperitoneal tumor, Ganglioneuroma, Supine hypotensive syndrome, Laparoscopic resection, Case report

## Abstract

**Background:**

Supine hypotension syndrome (SHS) has been reported to occur due to compression by a giant tumor such as ovarian tumor. We herein report a case of retroperitoneal ganglioneuroma with SHS treated with laparoscopic resection.

**Case presentation:**

The patient was an 11-year-old male with right-sided abdominal pain. He had a pale complexion and tachycardia while falling asleep. Computed tomography (CT) and magnetic resonance imaging (MRI) showed a giant mass lesion (60 × 35 mm) with compression of the inferior vena cava (IVC) and duodenum ventrally and the right kidney caudally. The IVC was flattened by mass compression. Abdominal ultrasonography (US) revealed narrowing of the IVC due to the mass and accelerated blood flow after IVC stenosis in the supine and left lateral recumbent position. His pale complexion and tachycardia while falling asleep was thought to be due to decreased venous return caused by the tumor compressing the IVC, resulting hypotension. 123I-MIBG scintigraphy revealed no abnormal findings. Tumor markers were normal. He was diagnosed with SHS due to a right adrenal gland tumor. The tumor compressed the IVC from the dorsal side, and hemostasis was expected to be difficult during bleeding. Therefore, a guidewire was inserted from the right femoral vein into the IVC for emergency balloon insertion during bleeding. A laparoscopic tumor resection was performed. A histopathological examination confirmed the diagnosis of primary retroperitoneal ganglioneuroma.

**Conclusions:**

The treatment of symptomatic retroperitoneal tumors requires a multidisciplinary approach.

## Background

Supine hypotension syndrome (SHS) occurs when a gestational uterus or mass compresses the inferior vena cava (IVC), thereby decreasing venous return to the right atrium and resulting in decreased cardiac output and hypotension. SHS is more likely to occur in pregnant women in late pregnancy when compressed by the uterus [1]. SHS has also been reported to occur due to compression by a giant tumor such as ovarian tumor [2]; however, there are no reports of SHS caused by tumor in children. We herein report a case of giant retroperitoneal ganglioneuroma with SHS treated with laparoscopic resection.

## Case presentation

**Fig. 1:**
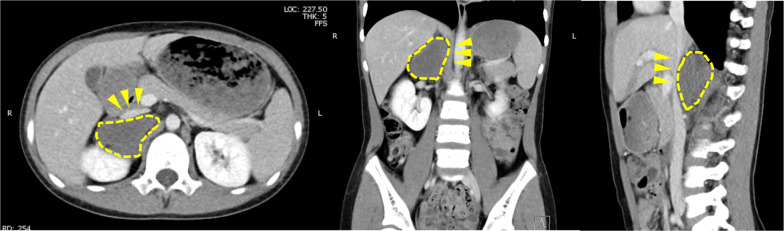
Abdominal CT showed that retroperitoneal tumor (dotted line) compressed IVC (arrowhead) and duodenum

**Fig. 2:**
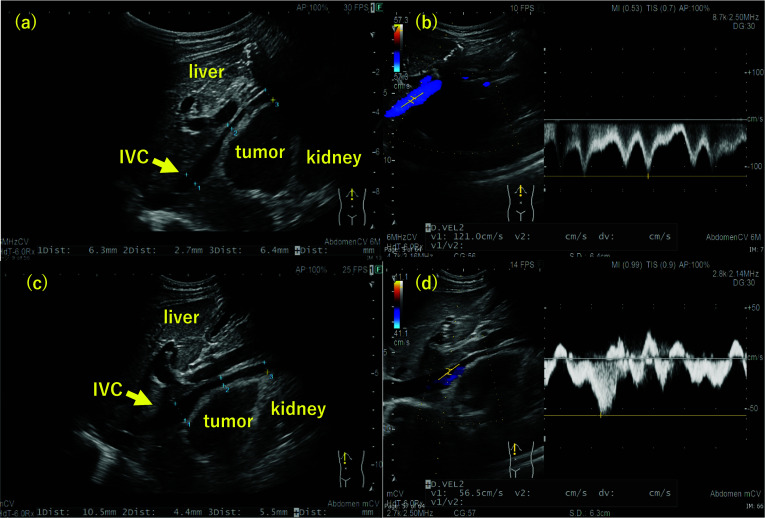
Abdominal US. **a**, **b** IVC was narrow and accelerated blood flow after IVC stenosis with supine position. **c**, **d** IVC was dilated and no accelerated blood flow in right lateral recumbent position

**Fig. 3:**
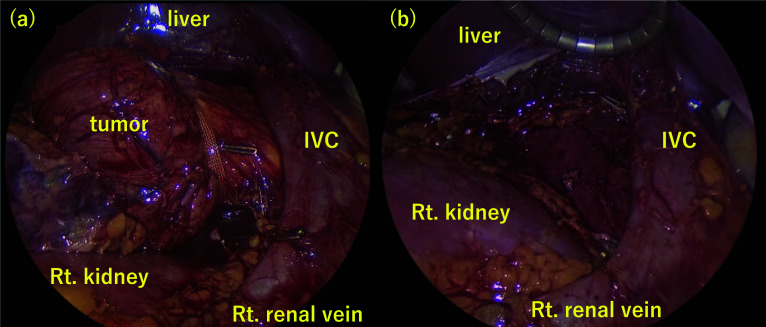
Operative finding. **a** Tumor was taped and resected with IVC dorsal view expansion. **b** The tumor was resected completely

**Fig. 4:**
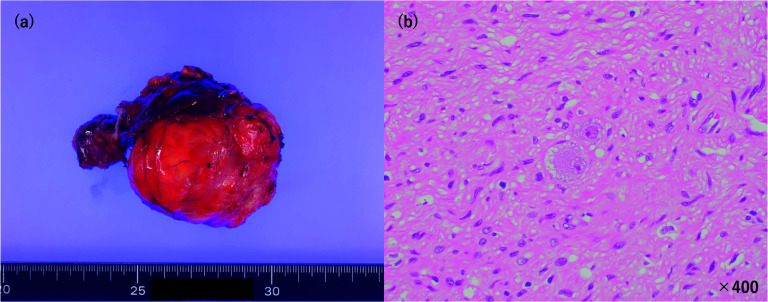
**a** Macroscopic findings. The size was 6.5 × 5.5 × 3.0 cm. The weight was 57 g. **b** Hematoxylin and eosin staining. The lesion was dominated by Schwann stroma and was surrounded by scattered ganglion cells. There was no continuity with adrenal tissue

The patient was an 11-year-old male. The patient presented to the outpatient clinic with right-sided abdominal pain. He had a pale complexion and tachycardia during waking. After a close examination, a retroperitoneal tumor was suspected, and the patient was referred to our hospital. His blood pressure in the standing was 100/71 mmHg. Computed tomography (CT) and magnetic resonance imaging (MRI) showed a mass lesion (60 × 35 mm) with compression of the IVC and duodenum ventrally and the right kidney caudally. The IVC was flattened by mass compression (Fig. 1). ^123^I-MIBG scintigraphy revealed no abnormal findings. Tumor markers, such as neuron-specific enolase (NSE), homovanillic acid (HVA), and vanillylmandelic acid (VMA) were normal. Abdominal ultrasonography (US) revealed narrowing of the IVC due to the mass and accelerated blood flow after IVC stenosis in the supine and left lateral recumbent positions. In the supine position, the flow velocity at the IVC stenosis was 78.8 cm/s, the proximal was 121.0 cm/s, and the distal was 128.1 cm/s. In the left lateral recumbent position, the flow velocity at the IVC stenosis was 124.8 cm/s, the proximal velocity was 210.5 cm/s, and the distal velocity was 71.8 cm/s. In the right lateral recumbent position, the flow velocity at the IVC stenosis was 58.6 cm/s, the proximal velocity was 56.5 cm/s, and the distal velocity was 39.2 cm/s. However, there was no blood flow acceleration in the right lateral recumbent position because the stenosis was released (Fig. 2). His pale complexion and tachycardia while falling asleep was thought to be due to decreased venous return caused by the tumor compressing the IVC, resulting in hypotension. He was diagnosed with SHS due to a right adrenal gland tumor. Although the tumor was considered unlikely to be malignant, surgical resection was planned because of SHS. During the waiting period for surgery, the patient was managed in the right lateral recumbent position and his pale complexion improved. The tumor compressed the IVC from the dorsal side and hemostasis was expected to be difficult during bleeding. Therefore, a guidewire was inserted from the right femoral vein into the IVC for emergency balloon insertion during bleeding. A laparoscopic tumor resection was performed (Fig. 3). The patient was placed in the left lateral recumbent position with a Free-Access S^®^ port with three 5-mm trocars inserted into a small 4-cm incision for tumor removal under the right flank. An additional 5-mm port was placed in the right intercostal space. The dissection proceeded dorsally, superior to the inferior margin of the tumor, and dissection between the IVC and the tumor was performed. The tumor was taped to avoid visual field expansion and grasping, and was resected up to the dorsal IVC. The patient was discharged on postoperative day 5. His blood pressure was 114/74 mmHg in the supine position. Histopathological examination confirmed a diagnosis of primary retroperitoneal ganglioneuroma (Fig. 4). Eight months after discharge, the patient is currently under outpatient observation without pallor, any other symptoms, or recurrence.

## Discussion

Gangliocytoma is a benign, slow growing, highly differentiated neurogenic tumor with a capsular membrane. The onset is usually 20 years of age. Preschool-aged children are less frequently affected [3]. The most common sites of occurrence were the posterior mediastinum (40%), retroperitoneum (30%), head and neck region, and adrenal glands [3, 4]. Ganglioneuromas are found incidentally in most cases and manifest as asymptomatic masses. The tumor can cause complications if it becomes large enough to press against the adjacent organs [5]. It can be followed up as a benign tumor; however, surgery should be considered in symptomatic cases. Ganglioneuroma is unresponsive to chemotherapy; therefore, surgical resection is the treatment approach. Careful dissection and preservation of these structures are paramount, especially in cases involving benign retroperitoneal masses [5].

In the present case, the patient was asymptomatic in the left lateral recumbent position for a short period of time and had his pallor during waking. It was thought that the prolonged left lateral recumbent position caused a gradual decrease in cardiac output due to decreased venous return, leading to hypotension. This case represents a rare manifestation of SHS, in which a retroperitoneal tumor compressed and stenosed the IVC in a sandwiched manner with the liver. The diagnosis posed challenges, as the stenosis findings varied with the patient's position. However, by observing blood flow in various positions, not only in the supine position, it was able to identify the IVH compression causing the symptoms. Additionally, determining the appropriate position that did not induce symptoms allowed for safe management during the preoperative period.

Laparoscopic surgery has been increasingly performed for adrenal lesions in recent years, even in cases of large tumors and severe adhesions, and is useful for reducing hospital stay and wound pain [6]. However, many retroperitoneal lesions are in close proximity to the large vascular system, which carries the risk of major bleeding during surgery and therefore requires emergency hemostatic measures. Neurogenic retroperitoneal tumors are often difficult to dissect from the dorsal neural tissue, so it is important to identify and prepare a visual field and recovery method for contingencies [7]. In the present case, the intraoperative bleeding was minimal. However, the IVC was deformed and the risk of intraoperative bleeding was high. Therefore, preparation for bleeding was implemented with the support of the cardiovascular team.

## Conclusions

Retroperitoneal ganglioneuromas associated with SHS are rare. Laparoscopic surgery can also be useful for retroperitoneal tumors in children, but it is important to prepare for contingencies due to the presence of a large vascular system, such as the IVC adjacent to the tumor. Therefore, treatment of symptomatic retroperitoneal tumors requires a multidisciplinary approach.

## Data Availability

Not applicable.

## Abbreviations

SHS: Supine hypotension syndrome
IVC: Inferior vena cava
CT: Computed tomography
MRI: Magnetic resonance imaging
NSE: Neuron-specific enolase
HVA: Homovanillic acid
VMA: Vanillylmandelic acid
US: Ultrasonography
